# Systemic analyses show that the biosynthesis and spatial distribution of fatty acids, triglycerides and lipids differed in male and female mice and humans

**DOI:** 10.1098/rsob.250198

**Published:** 2025-08-20

**Authors:** Samuel Furse, Samuel Virtue, Isabel Huang-Doran, Antonio Vidal-Puig, Davide Chiarugi, Philip C. Stevenson, Stuart G. Snowden, Albert Koulman

**Affiliations:** ^1^ Biological Chemistry Group, Royal Botanic Gardens, Kew, Surrey, UK; ^2^ Institute of Metabolic Science-Metabolic Research Laboratories, University of Cambridge, Addenbrooke’s Treatment Centre, Cambridge, UK; ^3^ Bioinformatics and Biostatistics Core, Wellcome-MRC Institute of Metabolic Science, University of Cambridge, Addenbrooke’s Treatment Centre, Cambridge, UK; ^4^ Max Planck Institute for Human Cognitive and Brain Sciences, Leipzig, Germany; ^5^ Biology Department, Royal Holloway, University of London, Egham, UK; ^6^ Natural Resources Institute, University of Greenwich, Chatham, Kent, UK

**Keywords:** lipids, biosynthesis, control, metabolism, differs, females

## Abstract

Recent work has shown that the prevalence and character of metabolic diseases differs between male and female mammals. This strongly suggests that the control mechanisms that govern, for example lipid metabolism, differ between the sexes. If true, a one-size-fits-all approach to treating metabolic disease will not be effective in all patients. We tested three hypotheses to understand how the lipid metabolism of male and female mammals may differ. First, whether endogenous fatty acid biosynthesis differed between tissues in the same male and female mice. Second, whether the system-level control of lipid pathways differed between the sexes. Third, whether lipid composition differs between males and females at a population level. We found that fatty acid biosynthesis was distinct in male and female mice across tissues. Systemic control of phospholipid and triglyceride metabolism also differed between the sexes. A human population showed that both phospholipid and triglyceride metabolism differed between males and females.

**Figure:**
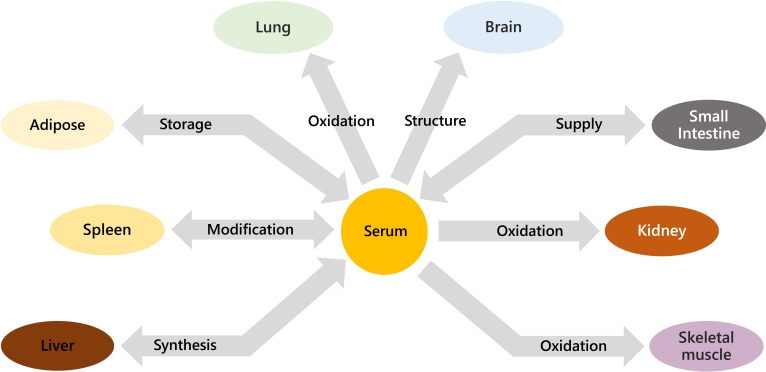

## 1. Introduction

Sexual dimorphism has been observed in the morphology of virtually all species in the animal kingdom [1]. Recent work has also found evidence for sexual dimorphism at the molecular level [2–7]. This includes molecular processes [8,9], disease states [10,11] and nutrition [12]. There is also evidence that dysregulation of metabolism is sexually dimorphic too, with the prevalence of metabolic disorders [13–19], the effects of exercise training to manage metabolic disease [20,21] and the response to drugs used to treat metabolic disease [22–24] differing between the sexes. Several studies suggest that lipid metabolism in particular is sexually dimorphic [25,26], consistent with the increasing number of studies showing that dysregulation of lipid metabolism is central to metabolic disease [27–30].

The evidence from studies of sexual dimorphism at the molecular level (molecular dimorphism) creates a picture that lipid metabolism is sexually dimorphic in health and disease. If true, treating metabolic disease the same way in males and females will lack efficacy in some or all patients. However, the current view of systemic lipid metabolism is that the differences between sexes in humans are due to gross body composition. This assumption is based on anatomical measures such as the volume of adipose being around 50% greater in females and the mass of skeletal muscle being about 38% greater in males [31]. However, lipid metabolism is primarily a molecular phenomenon and so it is difficult to extrapolate gross morphological differences to control of mechanisms at a molecular level. What is needed is a way to characterize the possible mechanisms that drive how and why male and female systems differ.

The molecular dimorphism studies done to date have been based on characterizations of individual compartments, separate from all the others in the same organism [2,3,7]. This has produced a picture that shows differences but does not characterize the relationships between compartments. The investigation of single tissue types is therefore limiting because metabolism is known to be a whole-body phenomenon with a variety of metabolic compartments (liver, hear, spleen, gut, etc.) all being connected and making different contributions [32–35]. The lack of a system-level analysis means that we do not currently know whether an apparent difference, for example in the liver, is unique to that compartment or representative of the whole system. This led us to suggest that a system-level analysis is needed to advance our understanding of how metabolism is controlled in male and female mice. This is particularly noticeable in lipid metabolism as the role and storage of lipids can differ considerably between compartments. Furthermore, in studies to date, typically male subjects were used in mammalian studies, meaning a comparison of the sexes was not possible [6].

The evidence for differences between male and female mammals, and the knowledge gap in understanding metabolism, led us to conceive a study based on a systemic analysis of lipid metabolism in male and female mammals. We noted two major experimental barriers to completing it. First, methods for acquiring appropriate metabolomics data from a considerable range of tissues are needed. In recent years, sample preparation and data acquisition from a variety of mammalian tissues has been reported [36–39], meaning that it is possible to acquire metabolomics data that describe a whole system. Second, a way of analysing the ‘big data’ that would inevitably emerge was required. Big datasets such as those generated by high throughput metabolomics typically contain many thousands of data points that cannot be readily interpreted by researchers from signals spreadsheets alone. However, computational tools for systemic molecular analyses for metabolomics data are less well developed. The KEGG pathway analysis is well established for small molecule interactions but is difficult to apply to lipid metabolism. Lipids have a variety of roles *in vivo*, there are often incremental differences between lipids of the same class and that the metabolic connections between lipids are not through a linear pathway. This required a computational tool that is not based on the substrate–enzyme–product model but a more reductionist approach in which the metabolites and where they are found in the system is used to assesses that system. A computational tool called Lipid Traffic Analysis (LTA) has been developed to overcome these experimental difficulties [40–43].

LTA is useful for systemic analysis of metabolic system because it has both quantitative and switch analyses. Quantitative analyses use RADAR plots to indicate which tissues show the greatest difference between groups across all compartments profiled, using Error-Normalized Fold Change to describe differences [42]. Switch Analyses show how the distribution of lipids differs between groups. Switch Analyses plot which lipids are present and where in the system (network) based on lipid types. ***A**-*type variables appear throughout the whole system. ***U**-*type variables are isolated metabolites, they only appear in one compartment for any one group. ***B**-*type variables appear in pairs of metabolically adjacent compartments. Plotting the presence or absence of these variables reveals the difference between groups and thus how lipid synthesis, degradation and transport differs between systems. This is distinct to measurements of metabolite flow or flux in which, for example, the rate of enzyme activity or transport are important. Traffic analysis plots show differences in the distribution of lipids, describing the number of variables of each lipid class through the system and thus show immediately which parts of the system and which lipid classes differ between groups. This reveals the patterns of differences in the control of lipid metabolism between groups. An example of this would be the accumulation of a set of PUFA-containing phosphatidylcholines (PCs) after feeding a PUFA [41]. The parts of the system that contrast the most sharply between groups are referred to as Control Points. Control Points are useful for characterising the mechanism(s) that drive the observed effects.

Control Points also characterize the general effects on the system. LTA has been used in hypothesis-driven studies to investigate metabolism and dysregulation of metabolism systemically [40–42], providing evidence for paternal nutritional programming [42], the relationship between a high fat diet and the risk of type II diabetes in female mice [44] and the effects of dietary intake of PUFAs in adult male mice [41]. Taken together, these studies show that the acquisition of lipidomics data and data analysis of networks is possible and insightful.

**Figure 1.**
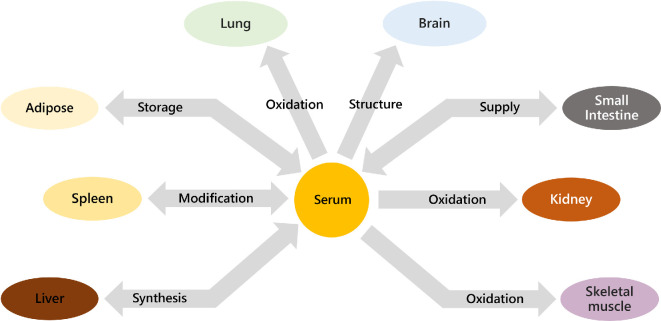
The biological network representing the metabolic system used in the mouse model used by Surma *et al.* [36]. The skeletal muscle sampled was vastus muscle.

The combination of evidence of molecular sexual dimorphism, a knowledge gap in understanding the difference in metabolism between the sexes (molecular dimorphism), and the emergence of techniques for collecting and analysing systemic data led us to test the hypothesis that the control of lipid metabolism is sexually dimorphic in adult mammals. We did this by testing three sub-hypotheses. First, whether endogenous fatty acid metabolism differed between male and female mice in organs from the same individuals. If true, this would show that differences in the control of lipid metabolism are observed in the biosynthesis of the most basic building blocks of lipids. Second, whether the distribution of phospholipids and triglycerides throughout the whole system was sexually dimorphic (i.e*.* whether control of pathways and distribution of lipids differed between the sexes). For this hypothesis, we used publicly available lipidomics data [36] and a new version of LTA, v3.0 [43]. This systemic analysis was based on the network of compartments across all major lipid highways (figure 1). Third, whether differences in lipid metabolism existed at a population level (i.e. whether molecular dimorphism is only visible in genetically similar groups of individuals). We used lipidomics data from the PROMIS study [45–48] to test this hypothesis. If this latter hypothesis were true, it would suggest more general trends in the difference between the sexes.

## 2. Results

### 2.1. Fatty acid composition of liver adipose and muscle in male and female mice

**Figure 2.**
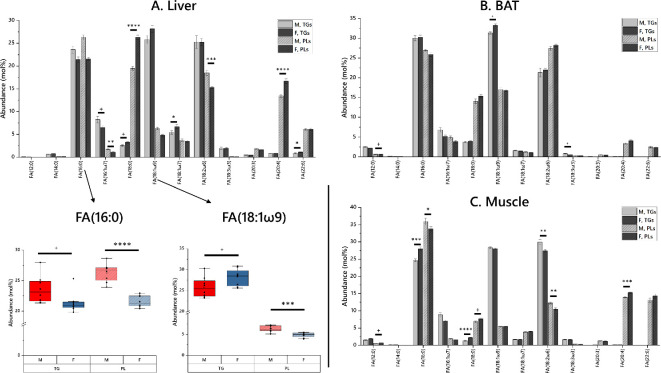
The abundance of fatty acids in mouse tissues from male (M) and female (F) mice. The lipid fraction was fractionated to glycerides (mainly TG) and phospholipids (PL). Mice were fed the same diet, data were acquired using GCMS. Error bars show propagated error. *<0.1, ^+^<0.05, **<0.01, ***<0.001, ****<0.0001.

We began by testing the hypothesis that the total fatty acid composition of a contrasting range of individual tissues from male and female mice fed the same diet differed. These sets of tissues came from the same individuals. Liver, brown adipose tissue (BAT) and vastus muscle were tested (figure 2A–C) as these tissues represent a contrasting set of roles and activity in lipid metabolism. In liver, we found that in females, C_16_ fatty acids were less abundant and C_18_ fatty acids were more abundant relative to males. The biggest difference was that steric acid was much higher in phospholipids of female than male mice. Females also had longer essential fatty acids in their phospholipids, with more FA(20:4) and less FA(18:2) (figure 2A).

The differences in the FA profile of vastus muscle were very similar to those observed in liver, albeit of smaller magnitude (figure 2C). Females had less FA(16:0) and FA(16:1), with more FA(18:0). Equally, females showed less FA(18:2) and more FA(20:4). Triglycerides in muscle showed a different pattern between the sexes, with more FA(16:0) and FA(18:0) in females and more FA(16:1) and FA(18:2) in males, suggesting that triglycerides in muscles were more desaturated in males than females.

Finally, in brown adipose tissue (BAT), there was more FA(18:0) in females but less FA(16:0) and FA(16:1) relative to males, i.e. a similar pattern to that of muscle and liver (figure 2B). However, in terms of *poly-*unsaturated fatty acids females contained more FA(20:4) (as seen in liver and muscle) but also contained more FA(18:2), suggesting the PL of BAT had generally more PUFA than in males. Triglycerides in BAT also showed a trend for longer saturated and *mono-*unsaturated fatty acids, however in this case, it was driven by reduced FA(16:1) and increased FA(18:1) in females relative to males.

Overall, a pattern emerged of females having longer fatty acids, favouring C_18_ species over C_16_ species in their saturated and *mono-*unsaturated and FA(20:4) over FA(18:2) in PUFA. Importantly, this pattern was observed across contrasting tissues from the same individuals, with several showing a >20% difference in abundance. We also note that the onward metabolism of these FAs was different in contrasting tissues (i.e. the FAs were found in different lipids, e.g. more FA(18:0) in was found in the PLs in liver and muscle, and more FA(18:1) was found in TGs in BAT). This suggested to us that as well as different biosynthesis of fatty acids in male and female mammals, different control of biosynthetic pathways of triglycerides and phospholipids existed, too. This led us to the hypothesis that the systemic lipid metabolism of male and female systems is organized differently. Testing this hypothesis required a set of metabolically connected tissues (i.e. including blood serum) from the same individuals, and is discussed in the following section.

### 2.2. Control of lipid metabolism in male and female mice

In order to test the hypothesis that the lipid metabolism of male and female systems is controlled differently, we used a dataset from a study by Surma *et al.* that comprised a variety of tissues collected from a single cohort of male and female mice that had been fed identical diets [36]. We plotted and used the spatial distribution of lipids through the system to determine how control of lipid biosynthesis pathways differed between groups. Data were plotted using the computational biology tool LTA v3.0 [40–43].

**Figure 3.**
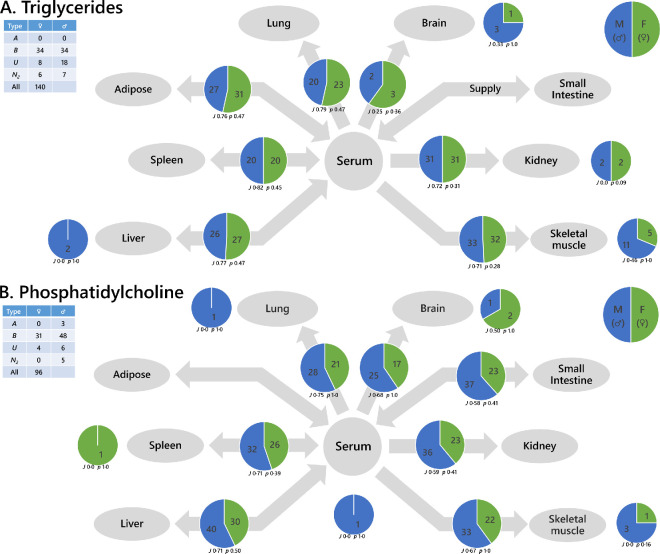
Traffic analyses of two lipid classes in outbred *M. musculus*: (A) triglycerides and (B) phosphatidylcholines. Larger pie charts (on arrows) represent variables found in the pairs of adjacent compartments (***B**-*type variables). Smaller pie charts represent isolated variables (***U**-*type). The table (inset) shows the total number of lipid variables of each type for the network. *J* represents the Jaccard-Tanimoto coefficient for the comparison, with accompanying *p*-value, as a non-parametric measure of the similarity between the variables identified in the two phenotypes for each comparison. The *p*-value shown represents the probability that the difference between the lists of variables for the two phenotypes occurred by random chance. TGs include all adducts of whole TGs and the DGs arising from in-source fragmentation of TGs during data collection.

The traffic analysis of triglycerides (TGs) in outbred mice (figure 3A) showed that there was a generally similar number of triglycerides in both sexes throughout the system, with a majority overlap (>70%) between the two. The triglycerides that were common to both sexes were ones commonplace in mammals; all those associated with *de novo* lipogenesis [49], TG(48:0, 48:1, 48:2, 50:1, 50:2) and polyunsaturated C_18_-containing TGs associated with omnivorous dietary intake, TG(54:3−6), were found in both sexes. However, the traffic analysis also showed there were two distinct control points (i.e. places in the system where the two groups differed markedly). There were a greater number of TGs in the brain and vastus muscles of male mice. The triglycerides that were unique to vastus (***U**-*type lipids) in this study were typically *mono-*unsaturated and odd-chain containing or C_20_-containing. Of the five ***U**-*type triglycerides shared between male and female mice, four were odd-chain containing and one C_20_. The remaining six triglycerides that were unique to male vastus muscle comprised odd-chain and C_20_ fatty acids but were slightly more unsaturated than the shared ***U***-type variables. This suggests that desaturase enzymes were more active in the male than female mice. In brain, the shared glyceride variable was DG(18:0/20:1), with DG(18:0/22:4) and DG(18:1/22:0) unique to male brains. This suggests that male brains contain a greater number of long-chain-containing lipids than do females.

The traffic analysis of PCs in outbred mice (figure 3B) showed that there are nearly 50% more isoforms of PC in male mice than female. There was generally a majority overlap between male and female mice, though female mice show some PCs that male mice do not. The male mice comprised a number of stearate- and oleate-containing PCs with C_20_ or C_22_ FAs that female mice did not. Several PCs were more widespread through the male system than the female. There was a control point in vastus muscle for this lipid class; three PCs unique to the vastus muscle were PC(14:0/20:4, 17:1/20:4, 18:2/22:5). These results show there is a greater range of polyunsaturated FAs in the phosphatidylcholine of male mice, and specifically in muscle tissue.

The same test was done in inbred mice, with similar results for the PCs but different ones for TGs (electronic supplementary material, figure S1A nd B, respectively). A wider traffic analysis, also plotting the sphingolipid, phosphatidylinositol and phosphatidylethanolamine classes identified other differences between in- and out-bred mice (electronic supplementary material, figure S1C). For example, the serum-spleen axis showed that TGs were distinct between male and female groups for inbred mice, whereas sphingolipids appear to show a more important difference in outbred mice. Several other disparities were found across the network (electronic supplementary material, figure S1C), indicating a wide-ranging set of differences in the control of lipid metabolism between in- and out-bred mice. This suggested to us that genetic diversity may also affect lipid metabolism and how it responds to challenges and in assessing differences in lipid metabolism between the sexes. Genetic diversity is wide in human populations and so we developed and tested the hypothesis that differences in lipid composition exist between males and females at a population level. This is discussed in the following section.

### 2.3. Differences in the supply of lipids in male and female humans

We used lipidomics data from human serum to test whether the differences between male and female systems were greater than the ranges within male and female systems. Data from the PROMIS study [45,46,48] were used for a random forest (RF) test to determine whether there is a significant difference in the lipid metabolism of males and females and to identify the variables driving this difference. The cohort (table 1) of male (4176) and female (1105) participants were split into two sets with the model trained on 1400 samples (700 male and 700 female) to avoid biasing our predictions and tested in the remaining 3476 males and 405 females of samples. The model generated was able to classify males and females in the test set with an accuracy of 73.7% (AUC = 79.7%), demonstrating that there appear to be differences in lipid metabolism between the sexes.

**Table 1.** Cohort data table. Cohort drawn from the PROMIS study [45,46,48]. BMI, body mass index.

		age				BMI			
	no.	mean	s.d.	min	max	mean	s.d.	min	max
cohort	5281	54.30	8.66	27.00	82.00	26.14	5.30	12.03	96.15
male	4176	54.12	8.70	27.00	82.00	25.68	4.69	12.03	96.15
female	1105	54.99	8.46	33.00	80.00	27.91	6.87	13.34	84.64

**Figure 4.**
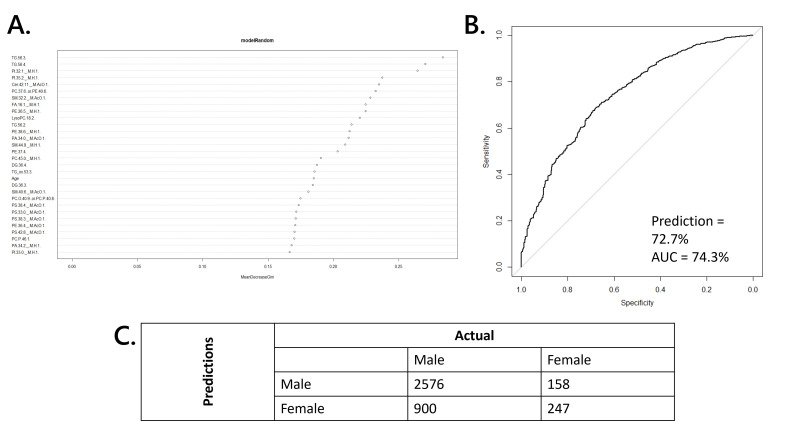
Plots showing the output of RF analysis of lipid profiles from males and females. (A) Plots showing relative importance to classification of lipid variables. (B) Receiver operating curve showing model performance. (C) Confusion matrix showing classification accuracy. Cer, ceramide; DG, diglyceride; FA, fatty acid; LPC, lyso-phosphatidylcholine, PA; phosphatidic acid; PC, phosphatidylcholine; PE, phosphatidylethanolamine; PI, phosphatidylinositol; PS, phosphatidylserine; SM, sphingomyelin; TG, triglyceride; TGoxo, oxidized triglyceride.

When examining the variables driving this model, we observed that BMI was ranked highly, something that could bias the result. We also regarded this of interest as BMI is a key risk factor in several metabolic diseases. We therefore generated a second model that corrected for BMI. This model classified the sexes in the test set with an accuracy of 72.7% (AUC = 74.3%), figure 4. The mean coefficient of variation for all lipid variables within genders was 43.9% and 16.9% for females and males, respectively, with the lower CV observed in men likely explained by the higher sample numbers, with a mean difference between sexes of 8.3%. The differences between the sexes were explained by both phospholipids and triglycerides, suggesting that the metabolism of both structural lipids and triglycerides differs between the sexes (table 2). Some of the phospholipids were more abundant in males and others less. By contrast, all the di- and triglyceride variables identified were less abundant in females than males. These variables, DG(36:3, 36:4) and TG(56:2, 56:3, 56:4) fall into the mid-range for size and unsaturation for glycerides in humans. With LPC(18:2), the glycerides form a set of lipids that are 10–15% less abundant in female humans.

**Table 2.** Lipid variables that distinguish the serum from male and female humans, generated using a random forest test, before and after correction for BMI. BMI, body mass index; CE, cholesterol ester; PA, phosphatidic acid; FA, fatty acid; LPC, lyso*-*phosphatidylcholine; PC, phosphatidylcholine; PE, phosphatidylethanolamine; PEO, phosphatidylethanolamine plasmalogen; PI, phosphatidylinositol; SM, sphingomyelin.

	uncorrected		corrected for BMI	
variable	*p*‐value	fold change (relative to males)	*p*‐value	fold change (relative to males)
Cer(42:11)			2.0×10^−16^	0.13
CE(16:1)	2.0 × 10^−16^	0.95		
CE(18:3)	2.0 × 10^−16^	0.97		
FA(16:1)	2.0 × 10^−16^	0.93	0.958	1
LPC(18:2)	2.0 × 10^−16^	1.05	2.0 × 10^−16^	0.89
LPC(20:4)	2.0 × 10^−16^	1.03		
PA(34:0)			1.5 × 10^−5^	1.11
PA(34:2)			9.3 × 10^−7^	0.86
PA(38:0)	2.0 × 10^−16^	0.95		
PA(40:2)	2.0 × 10^−16^	0.98		
PC(32:1)	2.0 × 10^−16^	0.96		
PC(34:3)	2.0 × 10^−16^	0.98		
PC(35:3)	2.0 × 10^−16^	0.98		
PC(37:4)			0.849	1
PC(37:6)	2.0 × 10^−16^	0.95	8.2 × 10^−11^	0.94
PC(45:0)			0.005	1.11
PC-O(31:2)	2.0 × 10^−16^	0.98		
PC-O(33:2)	2.0 × 10^−16^	0.97		
PC-O(36:5)	2.0 × 10^−16^	1.01		
PC-O(40:9)			4.6 × 10^−5^	1.25
PC-O(46:2)			1.9 × 10^−9^	0.85
PE(34:2)	2.0 × 10^−16^	0.96		
PE(36:4)	2.0 × 10^−16^	0.96	2.9 × 10^−10^	0.58
PE(36:5)			2.9 × 10^−13^	1.35
PE(37:4)	2.0 × 10^−16^	0.93		
PE(38:4)	2.0 × 10^−16^	0.97		
PE(38:6)	2.0 × 10^−16^	0.93	0.575	1.01
PE(39:0)	2.0 × 10^−16^	0.98		
PE(39:1)	2.0 × 10^−16^	0.98		
PE(41:2)	2.0 × 10^−16^	0.98		
PE(41:3)	2.0 × 10^−16^	0.99		
PI(32:1)	2.0 × 10^−16^	0.83	2.4 × 10^−11^	1.13
PI(35:2)			1.5 × 10^−5^	1.26
PS(33:0)			5.0 × 10^−6^	1.38
PS(38:3)			1.4 × 10^−5^	1.1
PS(38:4)			2.4 × 10^−5^	1.45
PS(42:8)			7.3 × 10^−6^	0.78
SM(32:2)			2.4 × 10^−7^	1.17
SM(34:2)	2.0 × 10^−16^	0.98		
SM(36:2)	2.0 × 10^−16^	0.97		
SM(40:6)			0.24	0.93
SM(44:9)			2.0 × 10^−16^	0.89
DG(36:3)			2.0 × 10^−16^	0.89
DG(36:4)			2.0 × 10^−16^	0.87
TG(56:2)			2.0 × 10^−16^	0.85
TG(56:3)	2.0 × 10^−16^	1.07	2.0 × 10^−16^	0.88
TG(56:4)	2.0 × 10^−16^	1.06	2.0 × 10^−16^	0.89
TGox(53:3)			2.0 × 10^−16^	0.89

## 3. Discussion

In this study, we report considerable differences in both the lipid composition and the control of lipid metabolism in male and female mammals. Lipidomics data from three independent datasets and two species (*Mus musculus* and *Homo sapiens*) showed these dissimilarities. Specifically, fatty acid synthesis showed similarly different patterns in contrasting tissues in male and female mice, reflecting changes in the control of systemic lipid metabolism.

In the study of the FA profile of individual mouse tissues, we found that the composition of endogenously-made fatty acids was different in male and female mice fed the same diet, supporting the hypothesis that endogenous fatty acid biosynthesis differed between tissues in the same male and female mice. Specifically, female mice had more FA(20:4) than the males, making it conceivable that the reduced levels of FA(18:1) relative to FA(18:0) in the phospholipids of females was a way to compensate for the increased membrane desaturation caused by the higher concentration of FA(20:4). These results suggest that membranes are fluidized by different unsaturated lipids in male and female mice. It is not clear why such compositional differences have arisen, however it does show that the distribution of polyunsaturated fatty acids differs between the sexes.

The second hypothesis, that the control of lipid pathways throughout the system differed between the sexes, was also supported. Lipid Traffic Analyses showed that the control of lipid metabolism differed between the sexes in terms of lipid pathway, fatty acid metabolism and whether mice were in- or out-bred. The differences between the sexes in triglyceride and phosphatidylcholine metabolism are striking because they show that although both energy storage and distribution (triglycerides) and structural lipids (phospholipids) are drawn from the same dietary fatty acid supply, the way the metabolism of the two are controlled differed between sexes. The traffic analysis of phosphatidylcholine (PC) showed that males had a greater number of and different PCs to females. However, TGs showed some local and some general differences, but also typically a different profile with a similar number of triglycerides. With the work at tissue level (figure 2) showing that endogenous biosynthesis of FAs differed considerably between the sexes and that the fatty acids in circulation differed between the sexes, this difference in the supply of fatty acids was reflected in the way that TGs and structural lipids are produced and implies important structural differences in the structure of the metabolism between male and female mammals in both a general and a local manner.

The evidence for tissue-specific differences in the traffic of triglycerides (figure 3A) supports the existing understanding that the accumulation of triglycerides in skeletal muscle differs in males and females, with a greater amount in females [50]. The systemic nature of the analysis done in the present study showed that triglycerides are indeed localized to skeletal muscle (interpreted as intra-myocellular fat) and thus this effect cannot be a general one. However, the systemic study done here showed no clear difference between the accumulation of TGs in the liver between livers of male and female mice, despite evidence that fat accumulation in the liver also differs between the sexes. This is intriguing as accumulation of triglycerides in liver is closely associated with dysregulated metabolism and is a serious risk factor in metabolic syndrome [51,52], a metabolic disease that is more common in male than female systems [50]. This suggests that the diet fed to the mice used in the present study was sufficient for male mice but slightly excess for the females (figure 3) [36]. This observation highlights the need to find out how selective the accumulation of triglycerides is and how that selectivity changes in response to challenges such as excess nutrition, i.e. for a sex-specific study of triglyceride traffic in non-alcoholic fatty liver disease (NAFLD). The interaction of the difference in accumulation of triglycerides between the sexes with excess nutrition also represents a knowledge gap for applications such as personalized medicine.

Evidence for a reorganization of triglyceride transport raises the question of the nature of the mechanism that drives the effect observed. Fatty liver occurs when the production or import of fatty acids into the liver exceeds the capacity for export or oxidation [53]. Changes to FA supply may drive ‘softer’ effects (i.e. effects that occur across several receptors in a number of tissues). A possible candidate for the type of signalling that might be affected by changes to FA synthesis and distribution is the activation of peroxisome proliferator activated receptors (PPAR). Jalouli *et al.* found that the sex-dependent differences in the expression of PPAR_α_ were driven by sex hormones derived from the pituitary gland [54], suggesting fundamental differences in lipid metabolism being driven directly by sex hormones.

The differences in PPAR_α_ activity driven by sex hormones contrast with the PPAR_γ_ activity that appears to have a similar effect on hypertension in both sexes in mice [55,56]. However, sex differences related to PPAR_γ_ have been observed in human T cells, alongside changes in the expression of PPAR_α_ [57], and in fetal mice in nutritional programming studies and parental diabetes [58,59] with no change in PPAR_α_. As sex differences in the expression of PPAR_β/δ_ have also been reported [60–62], there is a trend of a set of FA-agonized receptors that have a major role in controlling metabolism differing in expression between male and female individuals in health and disease. However, in a bimolecular pharmacological interaction, the substrate is as important as the receptor and so the effects observed may be partly due to changes in substrate. For example, Lohner *et al.* report in a meta-analysis that men have lower FA(20:4) and FA(22:6) than do women [26]. This implies that FA(22:6)-driven PPAR_γ_ agonism will be lower in men as they typically have less FA(22:6) *in circulo* [63]. However, current evidence suggests that PPAR_γ_ agonism is different in males and females, rather than substantially more or less. Taken together, the evidence surrounding FA activation of PPARs shows that both the receptors and the PUFA substrates that agonize them differ between the sexes. This leads us to suggest the general hypothesis that the summed effect of differences in expression of PPARs and the supply of FAs between the sexes is the basis for the different responses to metabolic challenges such as excess nutrition.

A key part of lipid supply in mammals is the lipids present in the circulation, as these represent the lipids that individual tissues are exposed to. In the present study, we used data from humans (the PROMIS study [45–48]) to test the hypothesis that differences in lipid composition exist between males and females at a population level. We found that both the structural lipids and the triglycerides found *in circulo* differed in male and female humans. Specifically, both unsaturated phosphatidylcholine and phosphatidylethanolamine were lower in females but triglycerides were higher. This is consistent with the wide-ranging changes seen in the systemic analysis of mice (*vide supra*), however BMI was also found to be an important factor in explaining the difference between groups. On correcting for BMI, we found that the fold change of TGs was inverted (>10% lower in females) and mixed results in phospholipids. This indicates that as well as sex, fat deposition is also important in explaining how whole-body lipid metabolism is controlled.

Whilst significant differences in lipid abundance were identified between males and females as shown by the results of the RF analysis (classification accuracy 72.9%) it is also clear that the mean variance within the genders (CVs *M* = 16.9% and *F* = 43.9%) is greater than the mean difference between genders (8.3%). This suggests that viewing the genders as monolithic populations is simplitic. There is significant overlap in the lipid abundances of males and females for even the most discriminatory variables suggesting that the observed differences are being driven by subgroups at the extremes rather than inherent differences in all individuals.

Taken in isolation, these results seen in the human population suggest that the lipid metabolism of males and females may fall on a continuum. However, at population level, other factors can have a bigger impact on lipid metabolism than sexual dimorphisms. It is also relevant to study the role of sex hormones in these differences and how these effect change over time. There are a variety of challenges of interest, excess nutrition, nutritional programming and exercise are three. A detailed understanding of both the baseline and the response to metabolic challenges together will inform nuanced applications such as personalized treatment for metabolic disease.

## 4. Conclusion

This study showed that there are myriad differences between the lipid metabolism of healthy male and female mammals. The profile of both chain length and unsaturation of FAs differs between male and female mammals and across contrasting tissues, suggesting that the needs of male and female systems for lipid metabolism differ, and even that some FAs are ‘replaced’ by others at tissue level. These tissue-level effects were also visible at system level, along with other effects such as which lipids and triglycerides the FAs are shunted into. These results showed that as well as different biosynthesis, the systemic control of lipid metabolism differs between the sexes. Human data showed that populations of male and female humans showed trends in the supply of lipids *in circulo*, supporting the observations about onward transport differing between the sexes and showing that there are trends in differences between the sexes that are greater than the diversity in populations. Taken together, the results from this study showed that there are baseline differences between the sexes that suggest the same metabolic challenge will have different effects in male and female systems. This hints that the effects of environmental stresses and even the treatment for apparently similar metabolic conditions may need to be different to fully treat male and female individuals.

## 5. Material and methods

### 5.1. Study design

This study was designed to identify how lipid metabolism differs between male and female mammals. Lipidomics data were used to measure the abundance of lipids in biological samples, and LTA [40–42] for systemic analyses where appropriate data existed. FA data from several tissues from mice was used to test whether there were basic differences between sexes, then a systemic analysis was done in mice. No such systemic analysis is possible in humans so a test of serum samples was undertaken. Existing data from Surma *et al.* and the PROMIS study were used [36,47], along with newly-generated data (figure 2).

### 5.2. Animal models

Mice were purchased from Charles River Ltd. and housed in a specific pathogen free facility with 12 h light and 12 h dark cycles (Light cycle 06:00 h to 18:00 h). All animals were studied at 24°C and a humidity of 55%. Standard laboratory chow diet (Research Diets D12450B, protein 20%, carbohydrate 70% and fat 10%) and water were fed *ad libitum* to both male and female mice used in this study. Tissues were collected after the animals (*n =* 8 per group) were euthanized by cervical dislocation (no anaesthetic was used). The animal models used to generate the publicly available lipidomics data used in this study are described in the original paper by Surma *et al.* [36]. Surma *et al.* used two mouse strains, Hsd:ICR (CD-1) and C57BL/6JOlaHsd for inbred and outbred mice, respectively, with at least three individuals in each diet-sex-strain group.

### 5.3. Human data

All human data came from the PROMIS study [45–48]. Table 1 shows the cohort data for this part of the study.

### 5.4. Lipidomics

Lipidomics data were drawn from two other studies. Data published by Surma *et al.* [36] was used for traffic analyses and data from the PROMIS study was used for RF analyses [25]. Novel fatty acid profiles were generated from dissected mouse tissues, using 8 mice per group.

### 5.5. Lipid extraction

#### 5.5.1. Whole lipid fraction

The lipid fractions were extracted from frozen tissue using a modified Folch method, described in detail elsewhere [64]. Briefly, the organic solvent mix (chloroform:methanol, 2:1, 1 mL, *v/v*) was added to weighed tissue samples liver (approx. 90 mg), muscle (approx. 100 mg) and Brown Adipose Tissue (30 mg) and WAT (approx. 100 mg). Internal standards were then added (see table 3)

**Table 3.** Internal standards added to 50−100 mg of tissue homogenized in chloroform/methanol. BAT, Brown Adipose Tissue; WAT, White Adipose Tissue.

tissue	TG(17:0/17:0/17:0)	TG(13:0/13:0/13:0)	PC(19:0/19:0)	PC(11:0/11:0)
liver	50 µg		500 µg	
muscle		125 µg		125 µg
BAT		125 µg		125 µg
WAT	5 mg			

Samples were homogenized with ceramic beads (MP biomedicals 6540−434, 5 Hz, 2 min, r.t.p.) and centrifuged (2 min, 21k × *g*). The organic solution was removed to a fresh phial and water added (200 µL mL^−1^ of organic solution). Samples were vortexed (5 min) and centrifuged (16 000 r.p.m., 10 min). The organic phase was collected and the aqueous phase washed once (chloroform, 700 µL mL^−1^ of the original solvent mix) and combined with the first organic phase. Samples were then dried (stream of gaseous nitrogen) and either separated into TGs and PLs (BAT, liver and muscle) or directly to preparing fatty acid methyl esters.

### 5.6. Separation of TGs and phospholipids

Lipid extracts from liver (approx. 90 mg), muscle (approx. 100 mg) and brown adipose tissue (30 mg) were resuspended (dried chloroform, 2 mL) and applied to an aminopropyl (NH_2_) solid-phase extract column (Agilent, UK). The elutate was collected, along with two subsequent fractions (chloroform, 1 mL; TGs), before eluting again (chloroform:methanol, 3:2, 2 × 1 mL). The appropriate fractions were combined and dried under nitrogen to give TG and PL isolates.

### 5.7. Preparation of fatty acid methyl esters

Lipid extracts were resuspended (chloroform, 375 µL) and diluted (methanol/BF_3_, 9 : 1, 500 µL) heated (85°C, 90 min) [64]. Samples were allowed to cool and washed (hexane, 1 mL) before being vortexed (2 min) and centrifuged (2000 r.p.m., 5 min). The organic solution was collected and dried.

### 5.8. Gas chromatography

Gas chromatography was performed on an Agilent 7890B GC connected to a 5977A MSD, using a Thermo Scientific TR-FAME column (length: 30 m, internal diameter: 0.25 mm, film size: 0.25 µm) with helium as carrier gas (2 mL min^−1^). Inlet and FID detector temperatures were set to 230°C and 250°C, respectively, MSD detector temperature was set at 230°C. Oven programme were as follows: 100°C 2 min, 25°C/min to 150°C, 2.5°C/min to 162°C, 162°C 3.8 min, 4.5°C/min to 173°C, 173°C 5 min, 5.0°C/min to 210°C, 40°C/min to 230°C, 230°C for 0.5 min. Results were presented and analysed as molar percentage of the total.

### 5.9. Lipid Traffic Analysis

Lipid Traffic Analysis code v3.0 [43] was used, recently updated from v2.3 [40,44,65]. Pie charts are used to represent the number of lipids (variables) on LTA plots. ***B***-type variables are shown by larger pie charts on the arrows between metabolically adjacent compartments. Isolated (***U***-type) variables are represented with smaller pie charts. A table (inset) is used to show the total number of lipid variables of each type for the network, including the ubiquitous lipids, i.e*.* lipids that are found throughout the network (***A***-type variables). In v3.0 of the LTA software, the code for the Switch Analysis was also updated to include alignment of lists in order to produce an additional output describing the traffic of each variable, for targeted analyses [43]. A novel lipid type was added for v3.0, referred to as ***N**_**2**_-*type, in addition to the existing ***U**-*, ***A***- and ***B***-type lipids. ***N**_**2**_*-type lipids are found in any two compartments and thus are distinct from *B*-type lipids. The full code for LTA v3.0 is available from https://pypi.org/project/lipidta/. Variables were regarded as present if they had a signal strength >0 in ≥66% of samples per group. Statistics for interpreting the meaning of these numbers are also presented; *J* represents the Jaccard–Tanimoto coefficient for the comparison, with accompanying *p*-value, as a measure of the similarity between the variables identified in the two phenotypes for each comparison. The *p*-value shown represents the probability that the difference between the lists of variables for the two phenotypes occurred by random chance.

### 5.10. Statistical methods

Univariate and bivariate statistical analyses were calculated in Microsoft Excel 2016. Graphs were prepared in Excel 2016 or OriginLab 2018. Calculations of Jaccard-Tanimoto coefficients (JTCs, *J*) and associated *p-*values [42] were used as a non-parametric measure of the distinctions between lipid variables associated with phenotype(s). The *p*-value associated with each *J* represents the probability that the difference between the lists of variables for the two phenotypes occurred by random chance, representing both the number of variables only found in either of the two groups and the order of the binary list. All lipidomic data were assumed to be unequally distributed and heteroscedastic and so the appropriate type of non-parametric test was applied.

## Data Availability

The externally generated data used in the present study were made publicly available by the authors, namely Surma et al. [36] and the PROMIS study [45–48]. The Python code used in the present study for Lipid Traffic Analysis v3.0 is publicly available through Github , offered with a CC-BY licence. Previous versions are available with the original papers (v1.0 [42], v2.3 [40,44,65]). Supplementary material is available online [66].
